# A Novel Photosensitizer for Lipid Droplet–Location Photodynamic Therapy

**DOI:** 10.3389/fchem.2021.701771

**Published:** 2021-06-14

**Authors:** Xiang Xia, Ran Wang, Yingqi Hu, WeiJian Liu, Ting Liu, Wen Sun, Jiangli Fan, Xiaojun Peng

**Affiliations:** ^1^ State Key Laboratory of Fine Chemicals, Dalian University of Technology, Dalian, China; ^2^ Ningbo Institute of Dalian University of Technology, Ningbo, China

**Keywords:** lipid droplet location, BODIPY, photosensitizer, photodynamic therapy, fluorescence imaging

## Abstract

Lipid droplets (LDs), an extremely important cellular organelle, are responsible for the storage of neutral lipids in multiple biological processes, which could be a potential target site for photodynamic therapy (PDT) of cancer. Herein, a lipid droplet–targeted photosensitizer (BODSeI) is developed, allowing for fluorescence imaging–guided PDT. Owing to the location of lipid droplets, BODSeI demonstrates enhanced PDT efficiency with an extremely low IC50 value (around 125 nM). Besides, BODSeI shows good biocompatibility and high photostability. Therefore, BODSeI is promising for droplet-location PDT, which may trigger wide interest for exploring the pathway of lipid droplet–location PDT.

## Introduction

As a basic unit of cells, subcellular organelles play significant and indispensable roles in diverse biological processes (Chorlay et al., 2019; Dalhaimer 2019; Soltysik et al., 2019; Cruz et al., 2020; Dubey et al., 2020). These subcellular organelles show different functionality and closely contact to form organelle interaction networks. LDs as highly dynamic organelles exert multiple and diverse effects in many cellular processes, including membrane synthesis, cellular energy homeostasis, trafficking, and protein degradation (Farese and Walther 2016; Goodman 2018; Xu et al., 2018; Nguyen and Olzmann 2019; Jin et al., 2020). They also interact with Golgi apparatus, mitochondria, endoplasmic reticulum, autophagic–lysosomal system, peroxisomes, and cytoskeleton (Klecker et al., 2017; Guo et al., 2021). Maintaining lipid metabolism homeostasis is essential for living cells. Thus, the destruction of LDs could disarrange cell metabolism and result in cell apoptosis, which could be a potential target for the treatment of diseases, such as cancer, one of the most lethal lesions.

Photodynamic therapy (PDT) is one of the most promising approaches for treating cancer, which requires three key elements including light, oxygen, and photosensitizers (PSs) (Chen et al., 2020; Jana et al., 2020; Tang et al., 2020; Wang et al., 2020; Xu et al., 2020). During treatment, PSs are activated upon light irradiation, which convert molecular oxygen to reactive oxygen species (ROS) to kill tumor cells. To date, various organic PSs have been developed, which are based on naphthalimide (Nguyen et al., 2019), cyanine (Ji et al., 2018), phthalocyanine (Abrahamse and Hamblin 2016), boron dipyrromethene (BODIPY) (Zhou et al., 2018; Chinna Ayya Swamy et al., 2020; Nguyen et al., 2020; Sitkowska et al., 2020), iridium, and ruthenium complexes (Wang K. N. et al., 2021; Lin et al., 2021; Yuan et al., 2021; Zhao et al., 2021). Among different dye scaffolds, BODIPY has been extensively studied as a potential PS candidate due to its excellent photochemical stability, high molar extinction coefficients, high quantum yield, and facile modification. However, pristine BODIPY demonstrates low triplet quantum yields; thus, promoting the intersystem crossing (ISC) of BODIPY derivatives is of great importance to achieve enhanced PDT effect (Xiong et al., 2018; Wang and Qian 2019; Radunz et al., 2020). There are several approaches that have been reported to enhance the ISC. These include constructing photoinduced electron transfer (PET) (Zhao et al., 2020) and spin–orbit charge transfer ISC systems in the PSs (Lei et al., 2020), as well as introducing heavy atoms to the molecules (Feng et al., 2020; Liu and Li 2020). Due to longer triplet lifetime and simple synthetic way, the approach that introduces heavy atoms like bromine (Br) or iodine (I) has been most commonly used in the design of PSs (Xue et al., 2018; Yuan et al., 2020). In fact, not only the ROS generation but also the cellular location of PSs affects the PDT efficiency. Until recently, although a number of PSs have been reported which demonstrate nucleus, mitochondrion, or lysosome location (Wu et al., 2015; Zeng et al., 2017; Li et al., 2019; Xu et al., 2019; Li et al., 2020; Wang R. et al., 2021), few PSs for LD location are also reported (Tabero et al., 2020; Zhang et al., 2020; Tan et al., 2021a; Tan et al., 2021b; Zhang et al., 2021). More importantly, the LD-targeted PDT mechanism is under deeper research. Consequently, it is crucial to develop an LD-targeted PS to explore a new way of PDT in cancer treatment.

**FIGURE 1:**
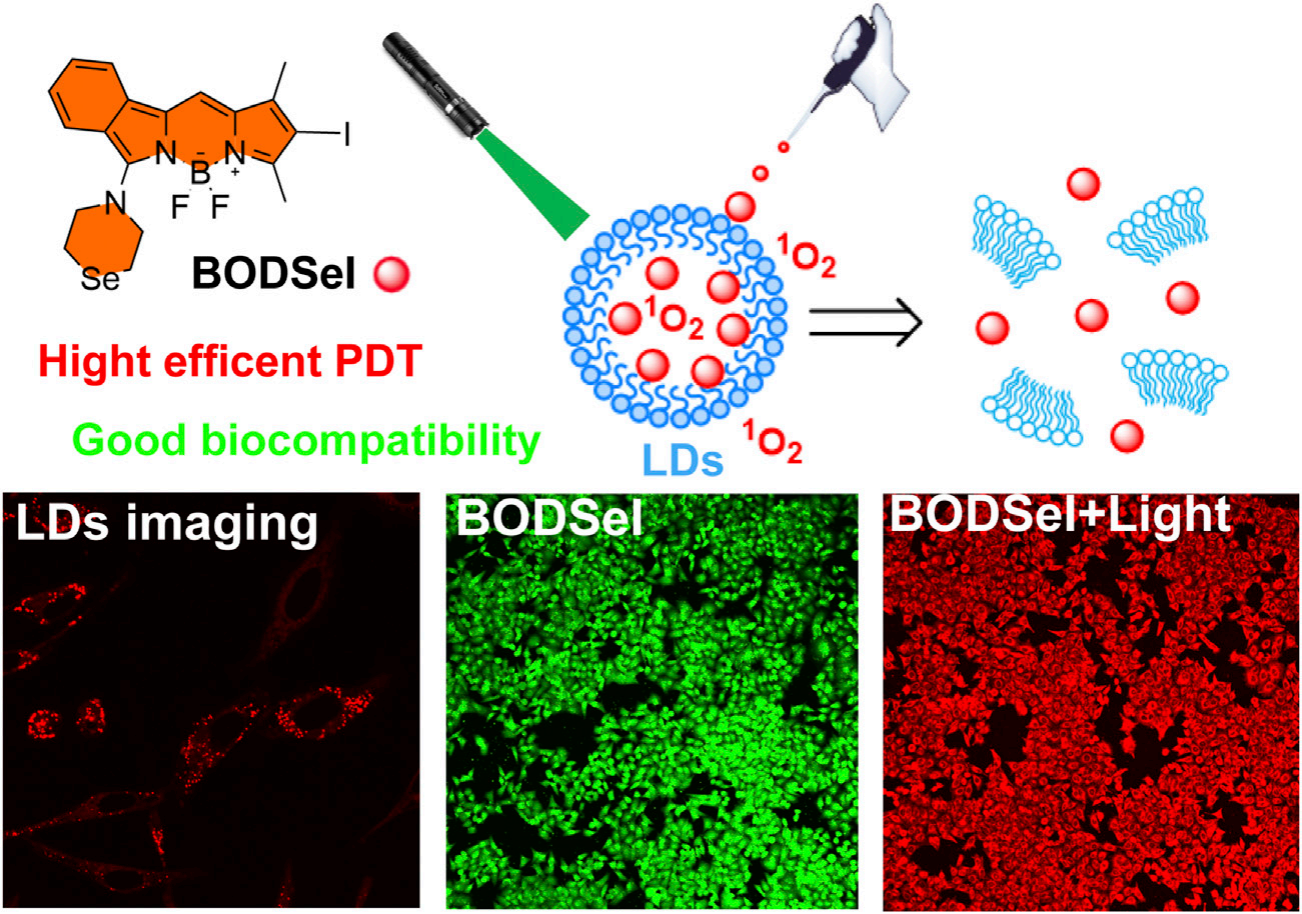
Schematic illustration of the LD-targeted strategy for photodynamic therapy.

Herein, based on BODIPY and the heavy-atom effect, we designed and synthesized a highly efficient PS BODSeI for lipid droplet–targeted PDT (Figure 1). The photochemical properties, bio-imaging, ROS photogeneration, photostability, and cytotoxicity with/without light irradiation of BODSeI have been investigated in this work. The cell imaging experiments demonstrated that BODSeI shows LD-targeting ability. In addition, the introduced reagent N-iodosuccinimide improved the ISC of BODSeI, resulting in a high single oxygen yield of 0.58, which is higher than that of the previously reported BODIPY PS (Li et al., 2017; Wang et al., 2019; Wang and Qian 2019). Importantly, the cell viability test suggested the superior performance of BODSeI in imaging-guided PDT with an extremely low IC50 value (around 0.125 nM) for three kinds of tumor cells, which could guide further design of PSs for LD-targeted PDT in cancer treatment.

## Materials and Methods

### Materials

All solvents and reagents were purchased from commercial suppliers and used without further purification. MTT [3-(4,5-dimethyl-2-thiazolyl)-2,5-diphenyl-2-H-tetrazolium bromide] and 1,3-diphenylisobenzofuran (DPBF) were purchased from Bide Pharmatech Ltd. Green fluorescence dye BODIPY493/503 lipid droplets were purchased from GlpBio Co. (Hoechst33342). LysoTracker Green DND 26 and MitoTracker Green FM were purchased from Life Technologies Co. Calcein-AM/Propidium Iodide (PI) Detection Kit was purchased from Beyotime Biotechnology Co. (China). Annexin V-FITC/Propidium Iodide (PI) Apoptosis Detection Kit was purchased from KeyGEN BioTECH Ltd. Human epithelial carcinoma HeLa cells, human breast cancer MCF-7 cells, and mouse breast cancer 4T1 cells were purchased from the Institute of Basic Medical Sciences (IBMS) of the Chinese Academy of Medical Sciences.

### Methods

^1^H-NMR and ^13^C-NMR spectra of all compounds were performed on a Bruker Avance III 400/500 spectrometer. Mass spectrometric (MS) data were carried out using Agilent 1260-6230 instruments. UV and fluorescence spectra were performed on a Lambda 35 UV-visible spectrophotometer (PerkinElmer) and a Varian Cary Eclipse fluorescence spectrophotometer (Serial No. FL0812-M018), respectively. Fluorescence imaging was performed on Olympus FV3000.

### Experiments

Compounds a and b were synthesized according to the reported method in Jiao et al. (2010).

#### Synthesis of BOD

Under Ar atmosphere, compound b (358 mg, 2 mmol), 2,4-dimethylpyrrole (206 μL, 2.0 mmol), and POCl_3_ (188 μL, 2.0 mmol) were added into a 50 ml round bottom flask containing 20 ml DCM, and the mixture was stirred for 0.5 h at room temperature. Then, triethylamine (5 ml) was added dropwise. Then, boron fluoride etherate (5 ml) was added into the above mixture, which was stirred for another 12 h. Finally, the mixture was washed with saturated sodium chloride and extracted by DCM. The solvent was evaporated in vacuum, and the resulting crude product was purified by silica gel column chromatography (hexane/EtOAc 5:1) to obtain BOD as a dark red solid (395 mg, 65%). ^1^H-NMR (400 MHz, CDCl_3_) δ = 7.79 (d, J = 8.2, 1H), 7.75 (d, J = 8.2, 1H), 7.54 (t, J = 7.5, 1H), 7.34 (s, 1H), 6.05 (s, 1H), 2.57 (s, 1H), 2.30 (s, 1H). MS: [M] calcd. for C_15_H_15_BN_2_ClF_2_: 304.08; found 304.10.

#### Synthesis of BODSe

BOD (400 mg, 13.5 mmol) and selenomorpholine [218 mg, 14.5 mmol, synthesized according to the reported method (Xu and Qian, 2019)] were added into a flask containing 10 mL DCM, which were stirred at room temperature, and the reaction was monitored by thin layer chromatography. After the reaction was finished, the solvent was removed, and the crude product was purified by silica gel column chromatography (hexane/EtOAc: 5:1–2:1) to obtain a deep red solid yield (330 mg, 60%). ^1^H-NMR (400 MHz, CDCl_3_) *δ* = 7.79 (d, J = 8.0, 1H), 7.75 (d, J = 8.3, 1H), 7.52 (t, J = 7.5, 1H), 7.32 (m, 2H), 7.06 (s, 1H), 5.93 (s, 1H), 4.43 (m, 4H), 3.06 (m, 4H), 2.49 (s, 3H), 2.26 (s, 3H). ^13^C-NMR (101 MHz, CDCl_3_) δ = 143.85, 137.36, 130.63, 128.94, 128.44, 127.97, 126.60, 125.79, 123.74, 119.49, 114.74, 109.71, 54.52, 17.93, 11.03. HRMS: [M] calcd. for C_19_H_20_BF_2_N_3_Se: 419.0884; found 419.0892.

#### Synthesis of BODSeI

BODSe (60.0 mg, 0.143 mmol) and N-iodosuccinimide (48.3 mg, 0.214 mmol) were dissolved in 10 ml DCM and stirred for 1 h at room temperature. After that, the mixture was extracted with saturated sodium thiosulfate solution to remove iodine. The crude product was purified by silica gel column chromatography (DCM:PE = 1:1) to afford BODSeI as a purplish red solid (35 mg, 45%). ^1^H-NMR (400 MHz, CDCl_3_) *δ* 7.78–7.76 (m, 2H), 7.56 (t, J = 7.3, 1H), 7.38 (d, J = 7.7, 1H), 7.04 (s, 1H), 4.48 (m, 4H), 3.04 (m, 4H), 2.52 (s, 3H), 2.21 (s, 3H). ^13^C-NMR (126 MHz, CDCl_3_) *δ* 153.66, 143.46, 140.54, 134.91, 131.47, 129.06, 127.70, 126.74, 122.18, 119.29, 116.89, 31.45, 29.71, 15.37, 13.67. HRMS: [M] calcd. for C_19_H_19_BF_2_IN_3_Se: 544.9850; found 544.9851.

## Results and Discussion

### Synthesis of BODSeI

**SCHEME 1:**
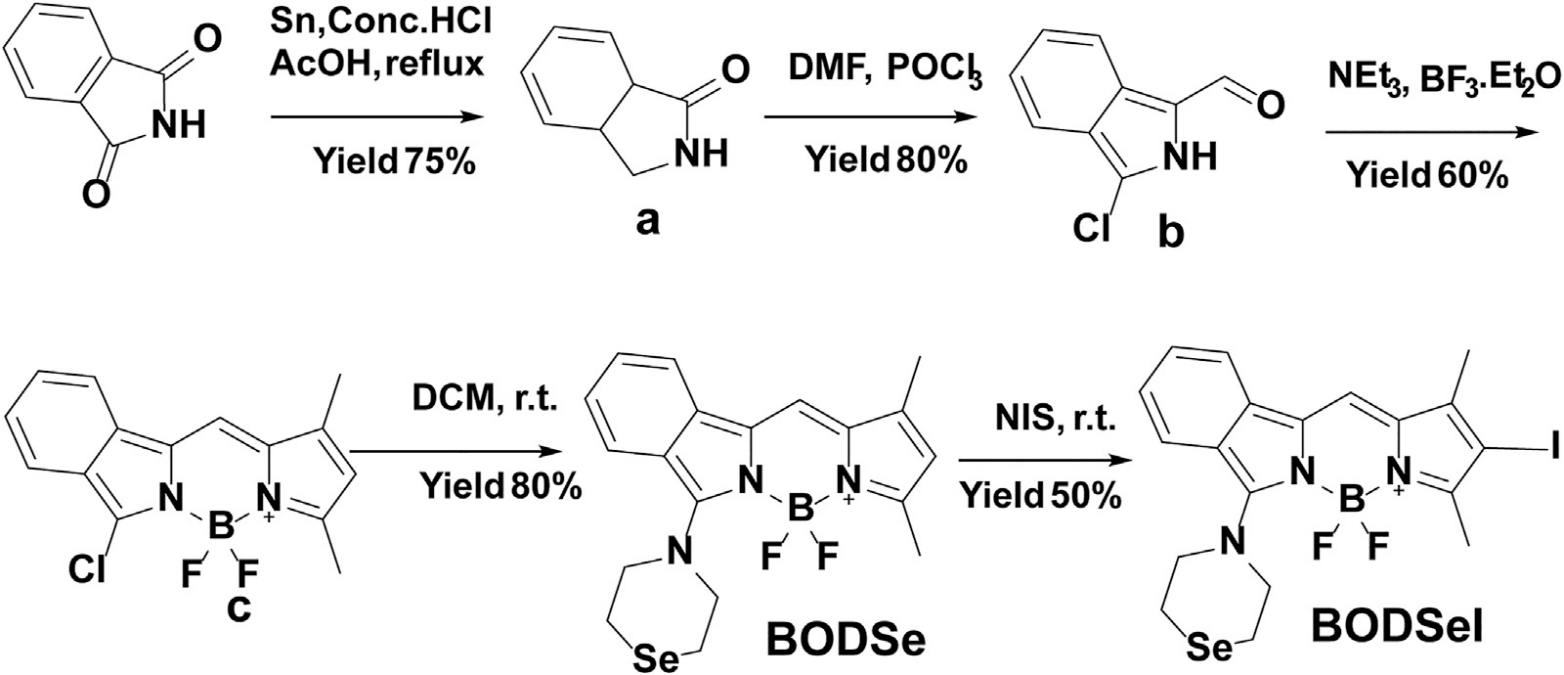
Synthesis route of BODSeI.

**FIGURE 2:**
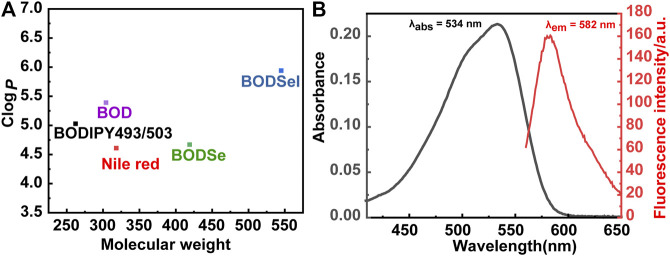
**(A)** Comparison of the molecular weight and Clog *P* of BODSeI, BOD, BODSe, Nile red, and BODIPY493/503. **(B)** Absorption and fluorescence spectra of BODSeI (1 μM) in DCM. Exicted at 535 nm, slits: 2.5/2.5 nm.

The synthetic route of BODSeI is presented in Scheme 1. The benzene ring was introduced to expand the conjugated system of BODIPY. As is widely acknowledged, heavy atoms like bromine and iodine, which enhance the spin–orbit coupling (SOC), promote the intersystem crossing (ISC) rate and increase the single oxygen production yield *via* the so-called heavy atom effect. Thus, an iodine atom was incorporated into the BODIPY scaffold to ensure high singlet oxygen production yield. In addition, a selenomorpholine group was attached to the conjugated system of BODIPY to adjust the lipophilicity and hydrophilicity for LD targeting. Clog *P* was defined as the calculated log *p* (n-octanol/water partition coefficient) value. For instance, Clog *p* > 5 is usually located in lipid droplet specificity. The Clog *p* values of BODSeI, intermediate BOD, BODSe, and the commercial lipid droplet fluorescent dyes (Nile red and BODIPY493/503) were compared. As shown in Figure 2A, compared with Nile red (4.62), BOD (5.39), BODSe (4.67), and BODIPY493/503 (5.02), Clog *p* of BODSeI is 5.94, indicating that the PS could possess good lipid droplet targeting. The absorption and fluorescence spectra of BODSeI in DCM was firstly investigated. As shown in Figure 2B, the maximum absorption and fluorescence emission wavelengths of BODSeI were 534 and 582 nm, respectively. Compared with BODIPY493/503, BODSeI demonstrated a larger Stokes shift and longer emission wavelength. We further investigated the influence of pH on the absorption and the fluorescence intensity of BODSeI. As shown in Supplementary Figures S2, S3, the absorption and fluorescence spectra of BODSeI did not show obvious change under different pH conditions (pH 4–9). Thus, BODSeI demonstrated good stability, which could be promising for LD imaging and thus could result in precious PDT *via* fluorescence imaging–guided treatment.

### ROS Detection in Solution and in Cells

**FIGURE 3:**
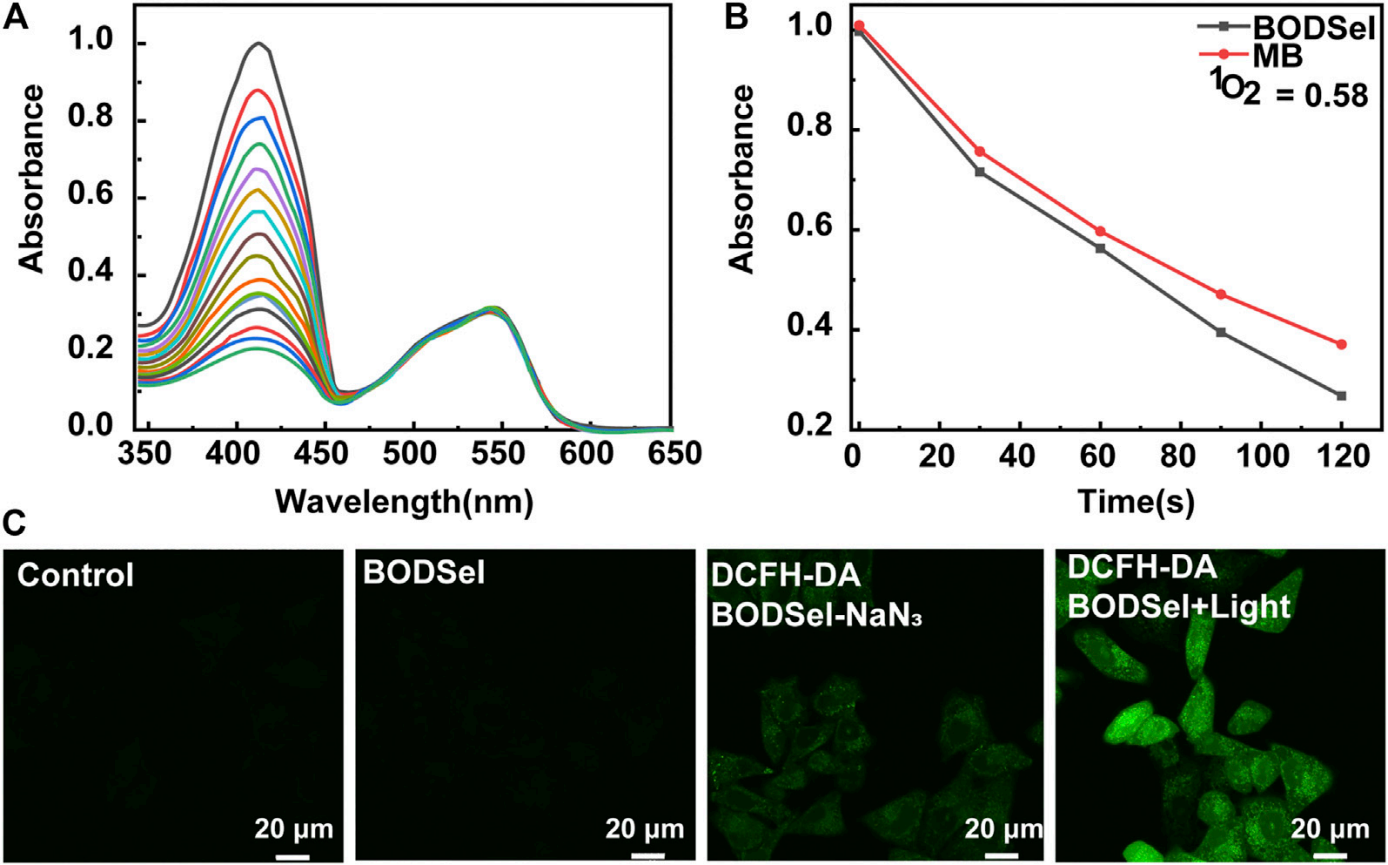
**(A)** Time-dependent photodegradation of DPBF with BODSeI in DCM. **(B)** DPBF degradation rates at different time points (the commercial PS; MB was used as a reference). **(C)** Fluorescence imaging of ROS detection with/without BODSeI (1 μM) and light irradiation (550 nm, 20 mw/cm^2^, 10 min) in MCF-7 cells [DCFH-DA (10 μM) was used as the fluorescence detector, and 50 μM NaN_3_ was used as the single oxygen quencher].

The ability of BODSeI to produce ROS was studied. Methylene blue served as a reference (Φ_△_ = 0.52 in DCM, absorption change of MB with DPBF is shown in Supplementary Figure S1), and DPBF was used as the single oxygen detector. With the irradiation time increased, the absorbance of DPBF decreased gradually (Figure 3A). Meanwhile, the absorbance peak of BODSeI almost unchanged, which indicated that BODSeI possesses high photostability without photobleaching. According to the DPBF degradation rate curves (Figure 3B), the single oxygen quantum of BODSeI was calculated to be 0.58, which is higher than those of the most previously reported PSs. Encouraged by the result of single oxygen generation in solution, the single oxygen production was examined in cells by using 2,7-dichlorodihydro-fluorescein diacetate (DCFH-DA) as the probe and NaN_3_ (50 μM/L) as the single oxygen quencher (Figure 3C). Only the group that coexisted with light irradiation and BODSeI showed strong green emission, indicating the ROS is generated. In addition, the group treated with BODSeI, light irradiation, and NaN_3_ showed low fluorescence emission, suggesting that the produced ROS is single oxygen.

### Subcellular Co-Localization

**FIGURE 4:**
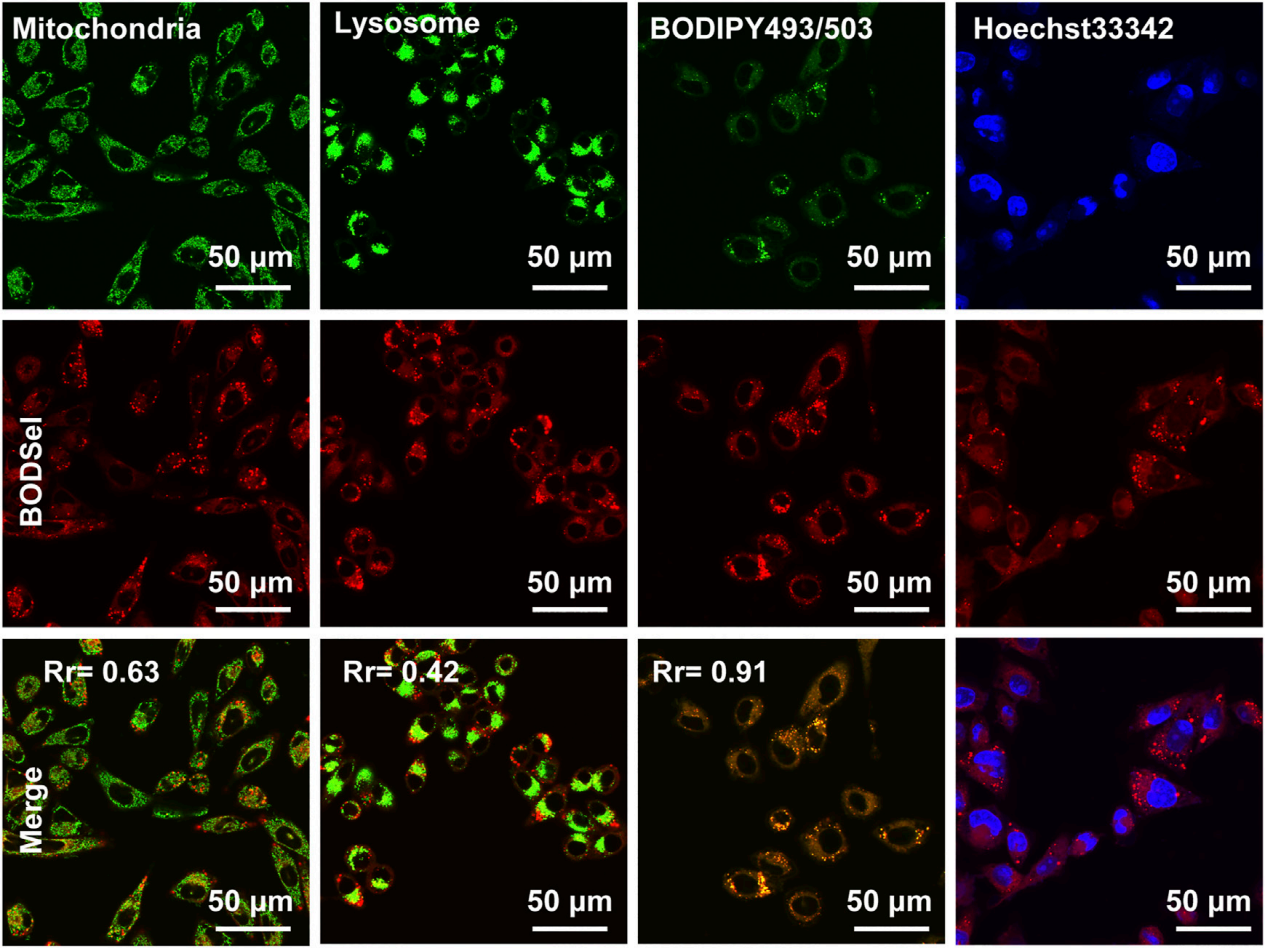
Co-localization imaging of MCF-7 cells. The cells were incubated with BODSeI at 37°C for 30 min in the serum-free medium, then treated with commercial fluorescence dyes at 37°C for another 30 min, and finally washed with PBS three times before confocal imaging. From top to bottom lines are commercial dyes, BODSeI, and merge channel. The concentration and excitation/emission wavelengths are provided in parentheses as follows: BODSeI (1 μM, λ_ex_ = 546 nm, λ_em_: 560–600 nm), MitoTracker Green (0.5 μM, λ_ex_ = 488 nm, λ_em_: 500–540 nm), LysoTracker Green (0.5 μM, λ_ex_ = 488 nm, λ_em_: 500–540 nm), BODIPY493/503 (0.5 μM, *λ*_ex_ = 488 nm, *λ*_ex_ = 500–530 nm), and Hoechst33342 (0.5 μM, λ_ex_ = 405 nm, λ_em_: 430–470 nm), respectively. Scale bar: 50 μm. The Pearson correlation coefficient (Rr) was calculated by Olympus FV3000 software Cell-Lens.

The effective inhibition of tumor cells was closely related with not only the production of ROS but also the distribution of PSs in cells. Thus, the subcellular co-localization experiments of BODSeI were studied, by comparison with commercial cell organelle location dyes including MitoTracker Green (mitochondria), LysoTracker Green (lysosomes), BODIPY493/503 (lipid droplet), and Hoechst33342 (nucleus). As shown in Figure 4, fluorescence imaging of BODSeI was distributed in cytoplasm and in accordance with the lipid tracker. The Pearson co-localization coefficient between BODSeI and lipid droplet commercial dyes was 0.91, which was higher than those of other organelles (lysosomes: 0.43, mitochondria: 0.63). The results demonstrated that BODSeI was mainly located in lipid droplets.

### *In Vitro* PDT Efficacy Evaluation

**FIGURE 5:**
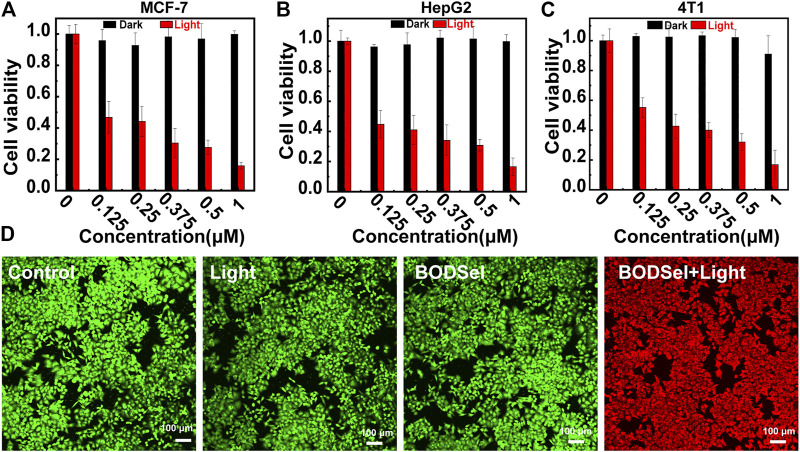
Cell viability of MCF-7 **(A)**, HepG-2 **(B)**, and 4T1 **(C)** cells incubated with different concentrations of BODSeI with or without light irradiation (550 nm, 20 mW/cm^2^, 10 min). **(D)** Live–dead cell staining with calcein-AM (green) and propidium iodine (red) after different treatments. Green light irradiation (550 nm, 20 mw/cm^2^, 10 min) was conducted after the cell was incubated with BODSeI (1 μM) for 30 min.

In order to assess the PDT effect of PSs, the cytotoxicity experiments of human liver carcinoma cells (HepG2), human breast cancer cells (MCF-7), and mouse breast cancer cells (4T1) were examined by a thiazolyl blue tetrazolium bromide assay. According to Figures 5A–C and Supplementary Figure S4, BODSeI and BODSe showed nearly no cytotoxicity under dark condition. More importantly, compared with BODSe, BODSeI demonstrated much enhanced cytotoxicity under light irradiation [xenon lamp equipped with a 550 nm filter (20 mW/cm^2^, 10 min)], and the viability of all these tumor cells decreased with the increasing dosage of BODSeI. Meanwhile, the half-maximal inhibitory concentration (IC50) of BODSeI for these tumor cells was around 125 nM, which indicated that BODSeI was able to kill tumor cells at low dose and showed a broad-spectrum anti-tumor character. To further visualize the effectiveness of phototherapeutics of BODSeI, live–dead cell staining was carried out through treatment with calcein-AM (green, live cells) and propidium iodide (red, dead cells). As expected, cells in the control, PS-only, and light-only groups showed strong green fluorescence (Figure 5D), suggesting that BODSeI demonstrated low dark toxicity and good biocompatibility. Almost all the tumor cells were killed in the experiment group of coexistence with BODSeI and light (550 nm, 20 mW/cm^2^, 10 min), which was due to the LD location and efficient ^1^O_2_ generation. Thus, BODSeI is promising for PDT *via* the location of LDs in cancer cells.

## Conclusion

In summary, we designed and synthesized BODIPY-based PS (BODSeI) for droplet-location PDT. The co-localization fluorescence imaging in subcellular experiments confirmed that BODSeI can specifically damage lipid droplets in cancer cells under light irradiation. Obvious phototoxicity was achieved in three different tumor cells including MCF-7, HepG2, and 4T1 with a lower IC50 (around 125 nM), which is lower than those of most commercial PSs. Easy acquisition, negligible dark toxicity, and good biocompatibility combined with efficient anti-cancer ability made BODSeI a potential phototherapeutic agent for further clinical studies. The design of LD location of PSs will be able to stimulate future design of more PS molecules with enhanced properties in cancer treatment.

## Data Availability

The original contributions presented in the study are included in the article/Supplementary Material, and further inquiries can be directed to the corresponding authors.
