# Supplementary material for: A Novel Photosensitizer for Lipid Droplet–Location Photodynamic Therapy
Source: Front Chem. 2021 Jun 14;9:701771. doi: 10.3389/fchem.2021.701771 (PMC8236597; doi:10.3389/fchem.2021.701771)
Supplement: Supplementary file 1 [file DataSheet1.docx]

Supplementary Material

**A Novel Photosensitizer for Lipid Droplet-Location Photodynamic Therapy**

Xiang Xia^1^, Ran Wang^1^, Yingqi Hu^1^, WeiJian Liu^1^, Ting Liu^1^, Wen Sun*^1,2^, Jiangli Fan*^1,2^, Xiaojun Peng^1^

**Cell experiments**

**Cell incubation and imaging**

Human breast cancer cells (MCF-7), Human liver carcinoma cells (HepG2), mouse breast cancer cells (4T1) were cultured in DMEM medium containing 10% fetal bovine serum (FBS) and 1% penicillin/streptomycin in a humidified atmosphere composed 5% CO_2_. Then cells were seeded on Cover glass-Bottom confocal dish and continuously incubated at 37 ℃ in a 5 % CO_2_ atmosphere for 24 h. Before the confocal laser scanning microscopy (FLUOVIEW FV3000, OLMYPUS) observation, the cells were washed three times with PBS buffer solution.

**Detection of ROS generation and cell apoptosis**

In order to evaluate the PDT effect of BODSeI in vitro, we used ROS 1,3-diphenlisobenzofuran (DPBF) as Singlet oxygen scavenger. Adjusted the absorbance of DPBF around 1.0 and the absorbance of BODSeI around 0.3. The ROS generated by excited BODSeI under xenon lamp with 550 nm filter and the dose of laser irradiation is 1 mW/cm^2^, record the UV-spectra with different time point. In vivo, the signals of intracellular ROS generation were detected by DCFH-DA (10 μM). Cells seeded in confocal laser scanning microscopy (CLSM)-special cell dishes were divided into control, only BODSeI without light, BODSeI (1 μM) with NaN_3_ (50 μM, as ^1^O_2_ quencher) and light，BODSeI (1 μM) with light. Cells were co-incubated with 10 μM DCFH-DA for 20 min at 37 ℃ before imaging DCFH-DA was excited at 488 nm.

The signal of cell apoptosis induced by intracellular ROS generation was also observed through monitoring confocal fluorescence of Calcein-AM/PI. Cells seeded in CLSM-special cell dishes were divided into control and other experimental groups. Control group, light only and BODSeI (10 μM) only were incubated with AM (2 μM) and PI (4.5 μM) without light irradiation 20 mins before imaging. BODSeI (10 μM) with light group was irradiation by xenon lamp with 550 filter (20 mW/cm^2^, 10 min), the fluorescence imaging was performed 4 h later.

**Cytotoxicity experiments**

Cell viabilities were evaluated through MTT assays. Mitochondrial dehydrogenases in living cells can reduce MTT (3-(4,5-dimethylthiazol-2-yl)-2,5-diphenyltetrazolium bromide) to formazan crystals. Complete medium (10% FBS, 100 μL) containing cells were seeded per well in the 96-well culture cluster (Costar) at a density of 6 × 10^4^ cells/mL. After cell attachment for 24 h, the cluster was then washed with 100 μL PBS/well. The cells adhered on the cluster were firstly cultured in complete medium with 0, 0.125, 0.25, 0.375, 0.5, 1 μM **BODSeI** for 0.5 h. Cells in the above experimental groups were irradiated by xenon lamp with 550 nm filter (20 mW/cm^2^, 10 min) then were further cultured for another 24 h. Cells in culture medium without **BODSeI** culture and laser irradiation were used as the control group. Both control and experimental groups were statistically analyzed in six replicated wells for increasing accuracy. After 24 h incubation, MTT (10 μL, 5 mg/mL) prepared in PBS was added to each well in the 96-well culture cluster for detecting cell viability and the culture cluster was incubated at 37 °C in a 5% CO_2_ humidified incubator. After 4 h culture, the medium was then carefully removed, and the purple crystals were dissolved in 100 μL DMSO per well. Considering that cell viability is proportional to the absorbance of formazan, which can be detected in a microplate reader, cell viability of experimental groups is expressed as an optical density percentage of formazan relative to the control group.

**Subcellular location assays**

The investigations of **BODSeI** subcellular localization were realized by the co-localization assays with commercial organelle dyes (Mito-Tracker Green (mitochondria), LysosomeTracker Green (lysosomes), BODIPY493/503 (lipid droplet) and Hoechst33342 (nucleus). **BODSeI** (1 μM, 20 min) and the commercial organelle trackers mentioned above were successively added to the same cell dish and cultured with cells at 37 °C for different duration in culture medium. The culture concentrations and working procedures of commercial trackers can be acquired from production instruction books provided by reagent companies. After incubation, cells were washed with PBS three times, and 1 mL serum-free medium was finally added before imaging. Cells were imaged with a 60 × oil-immersion objective lens in an inverted-type scanning microscope (Olympus FV3000). To avoid the cross-color interference, the fluorescence of **BODSeI**, and commercial fluorescence dyes were excited and collected in the different phases of confocal laser scanning microscope. The laser wavelengths to excite **BODSeI**, mito-tracker, lysosome, lipid droplet and Hoechst 33342, and the corresponding collection ranges of fluorescence emission are separately 546 nm (560–600 nm), 488 nm (500–540 nm), 488 nm (500–540 nm), 488 nm (500–530 nm), 405 nm (430–470 nm).


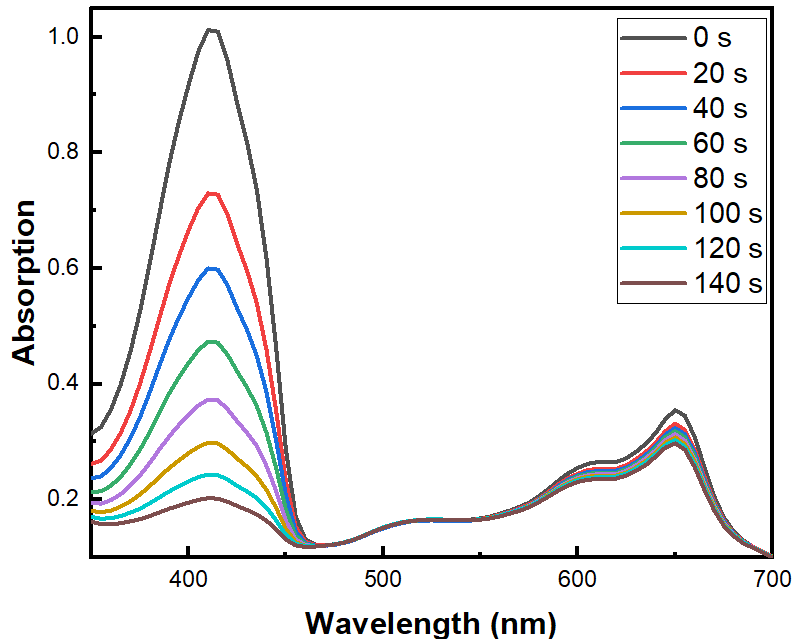


Figure S1, Time-dependent photodegradation of DPBF with MB in DCM.







Figure S2, effect of the pH on the UV(a) and fluorescence (b) of BODSeI in PBS buffer solution. The slight change of absorption maybe attributed to errors in the preparation of the test solution.


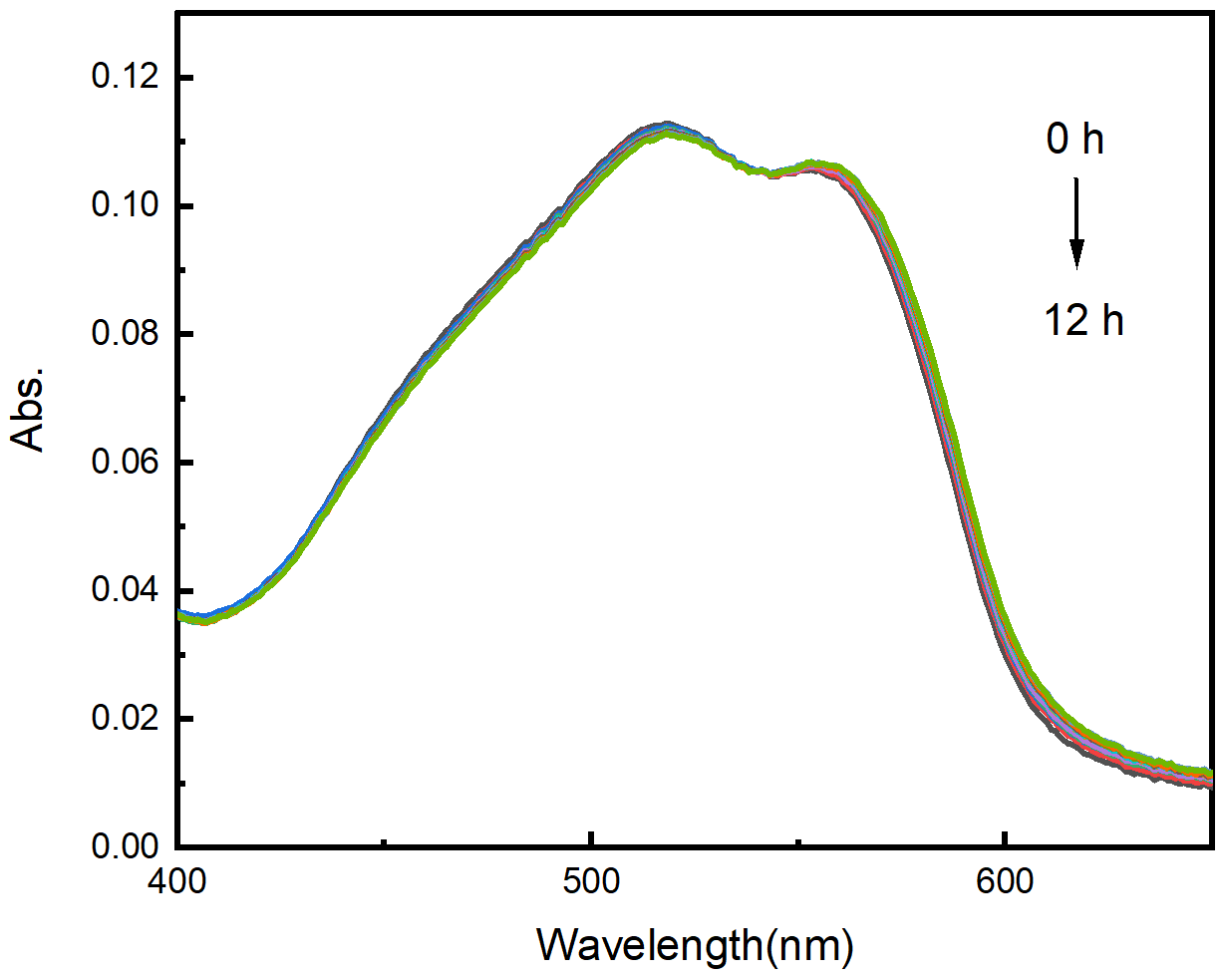


Figure S3, the UV spectrum of BODSeI in PBS buffer solution under different time.


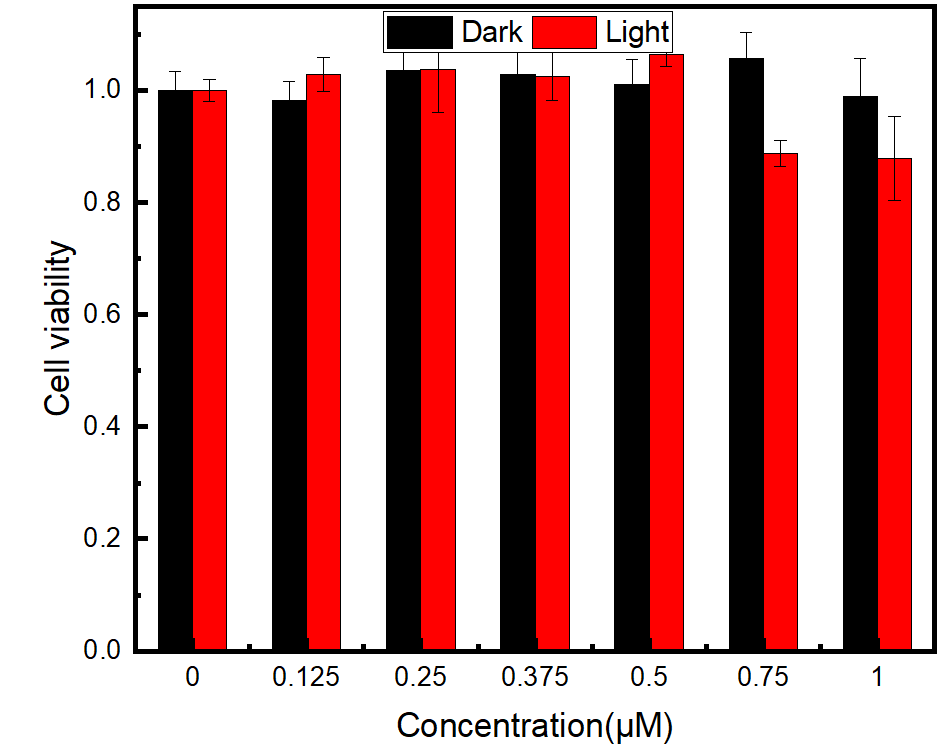


Figure S4, Cell viability of MCF-7 incubated with different concentrations of BODSe and iodine (Sodium iodide as a source of iodine) with or without light irradiation (550 nm, 20 mW/cm^2^ 10 min).


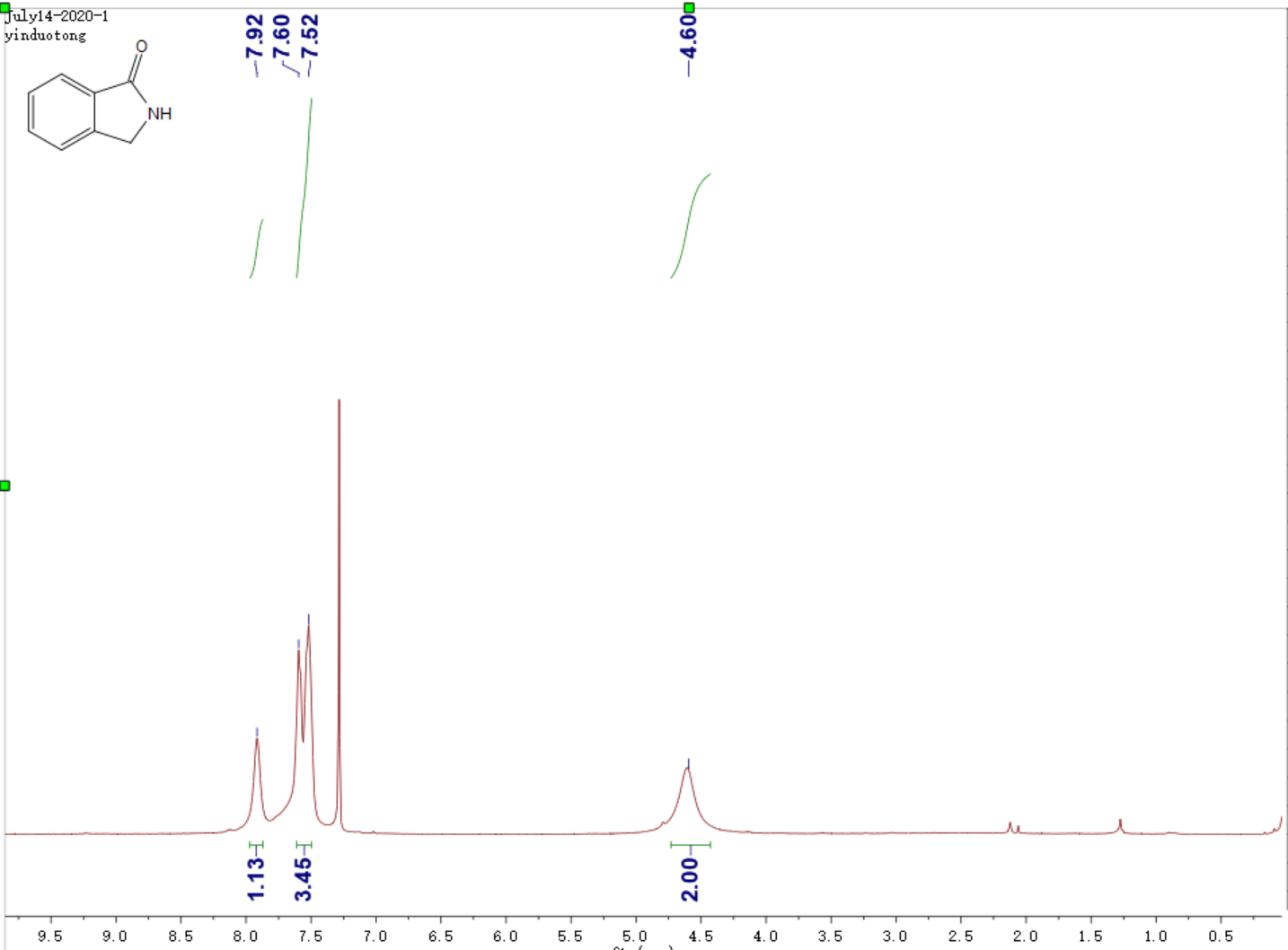


Figure S5, ^1^H-NMR spectrum of a in CDCl_3_


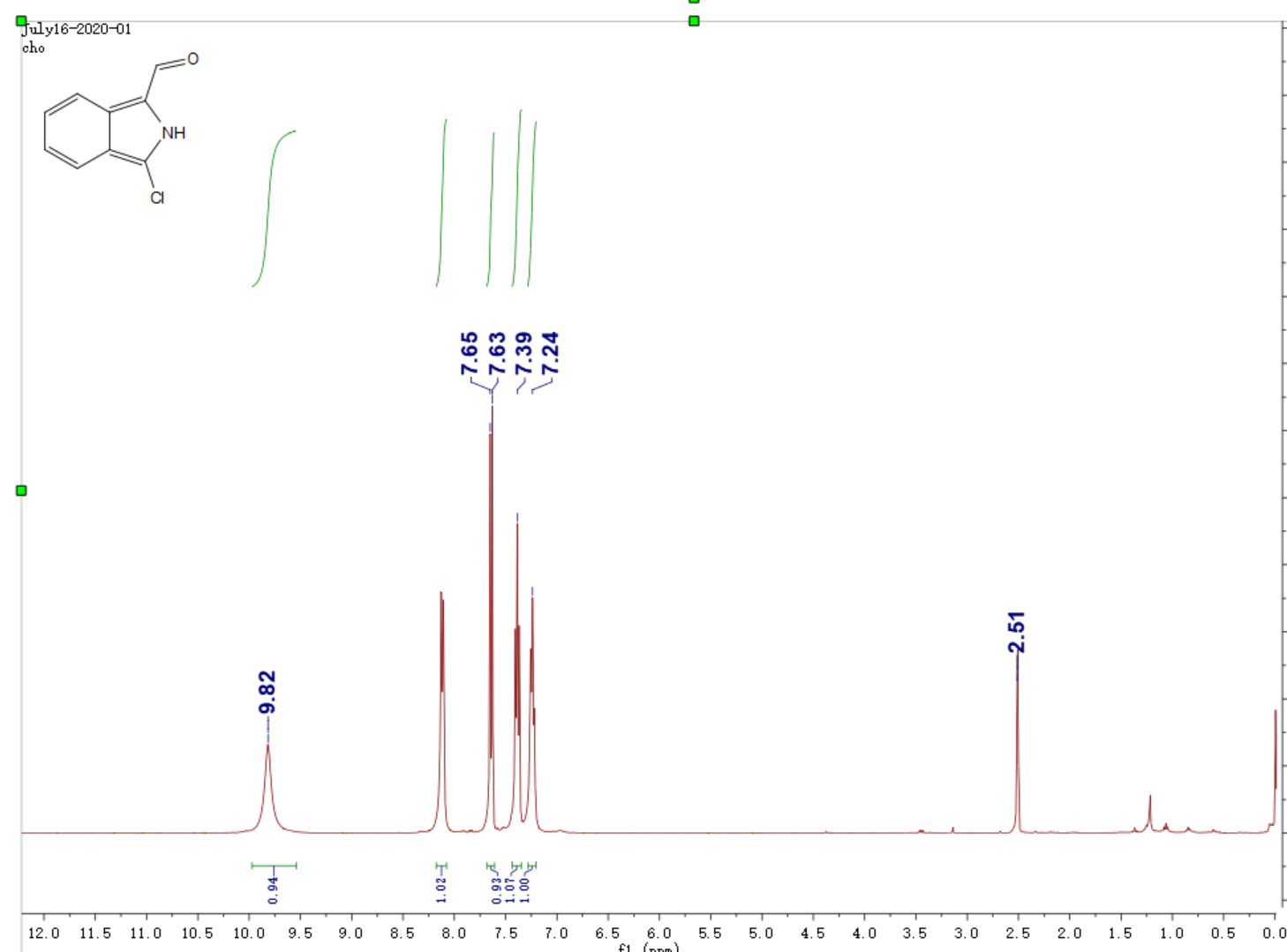


Figure S6, ^1^H-NMR spectrum of b in DMSO-d_6_


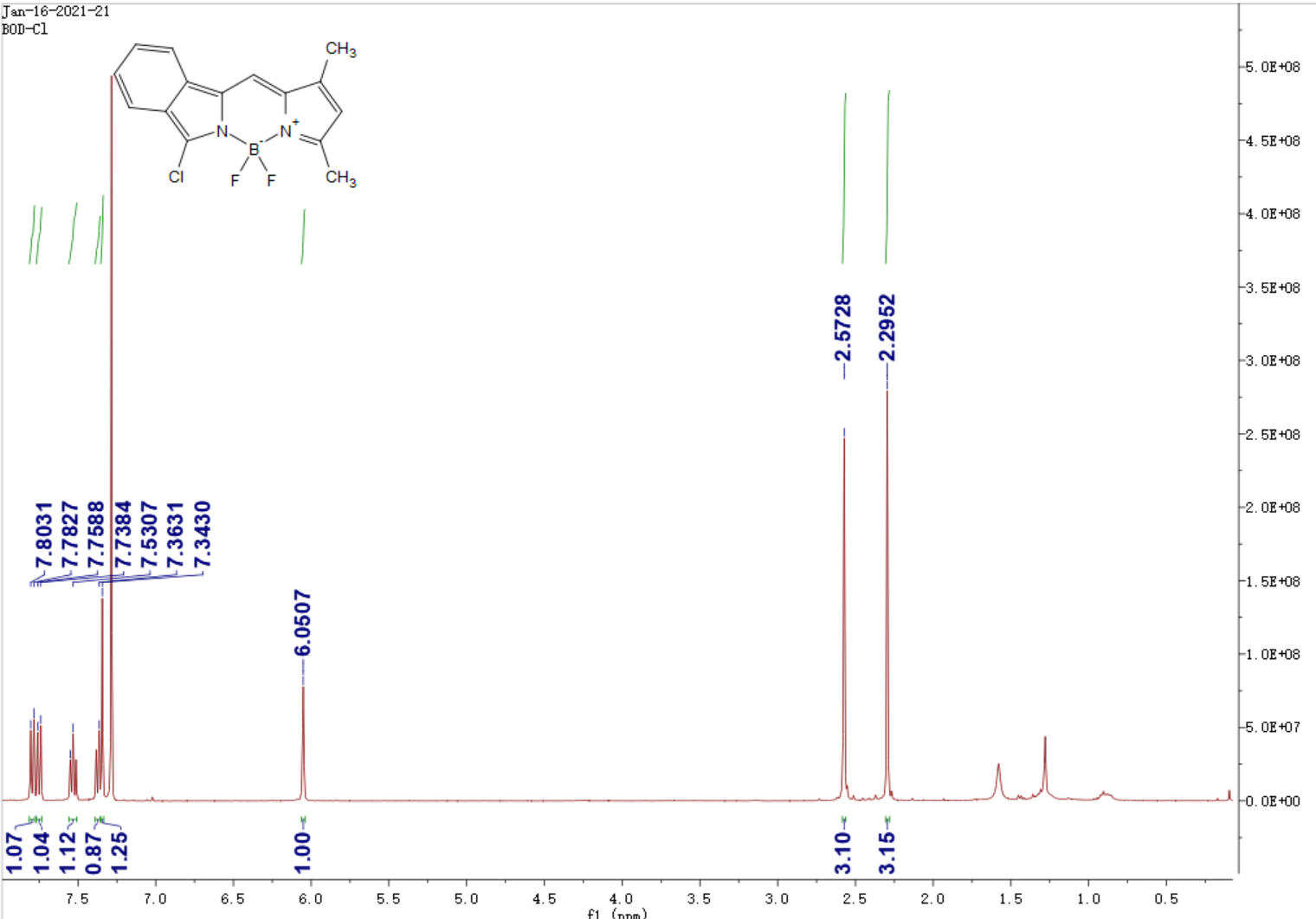


Figure S7, ^1^H-NMR spectrum of BOD in CDCl_3_

Figure S8, mass spectrum of BOD calcd. C_15_H_15_BN_2_ClF_2_: 304.1; found 304.1


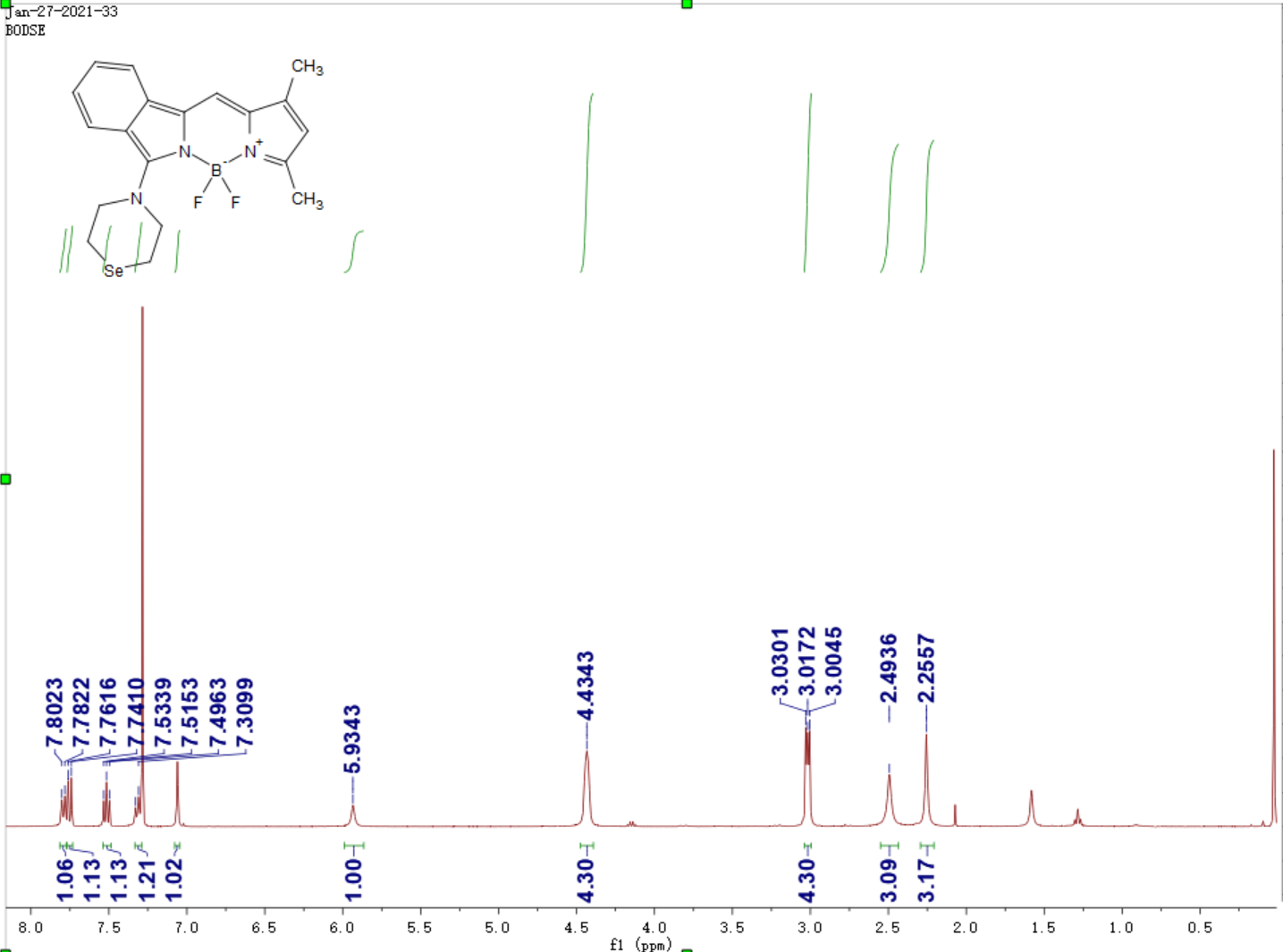


Figure S9, ^1^H-NMR spectrum of BODSe in CDCl_3_


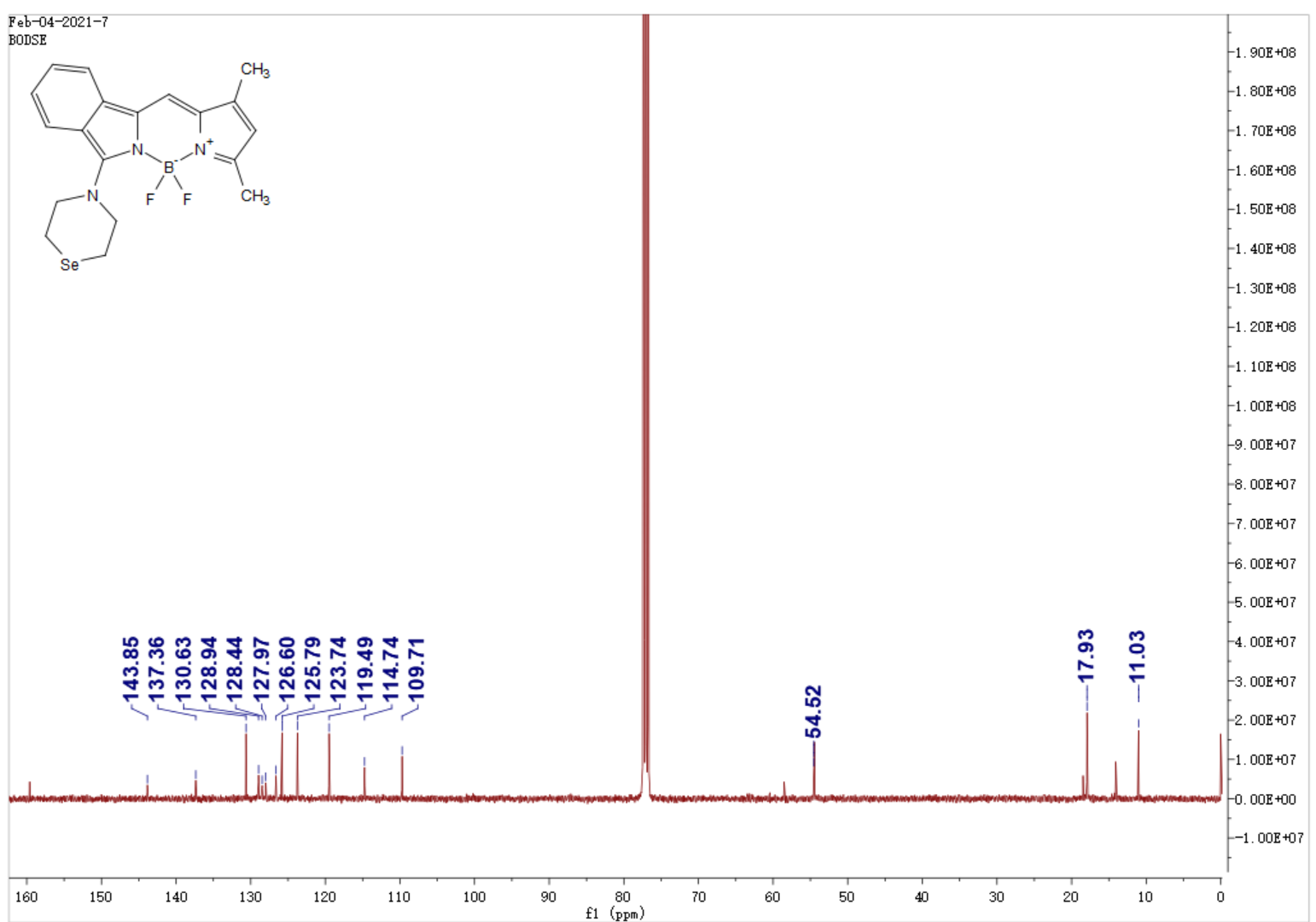


Figure S10, ^13^C-NMR spectrum of BODSe in CDCl_3_


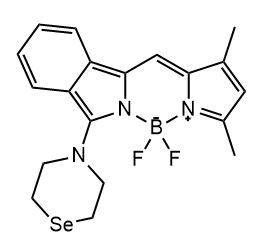


Figure S11, mass spectrum of BODSe calcd. [M], C_19_H_20_BF_2_ N_3_Se: 419.0884, [M+Na],442.0776; found [M]:419.0892, [M+Na]:442.0777.


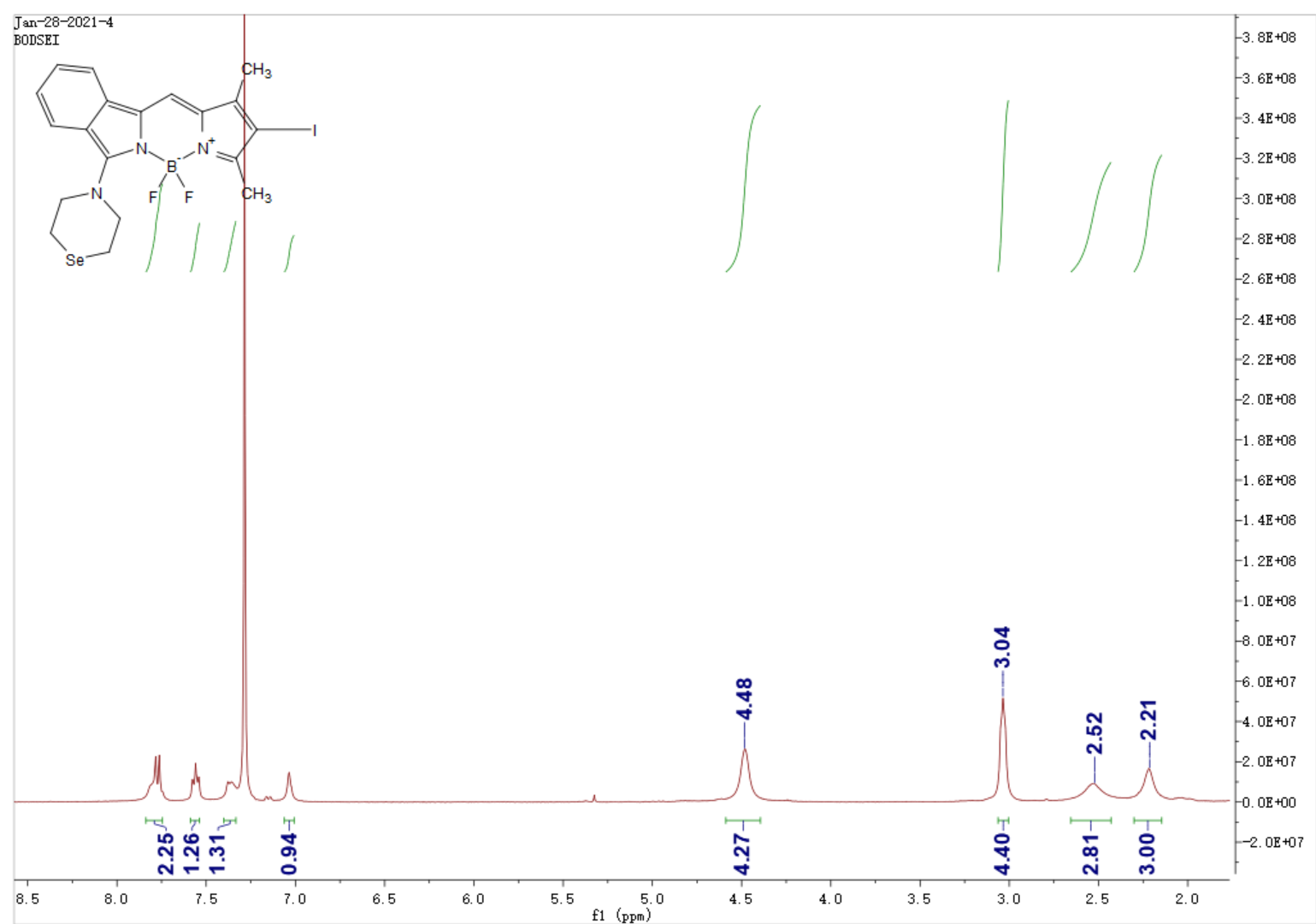


Figure S12, ^1^H-NMR spectrum of BODSe in CDCl_3_


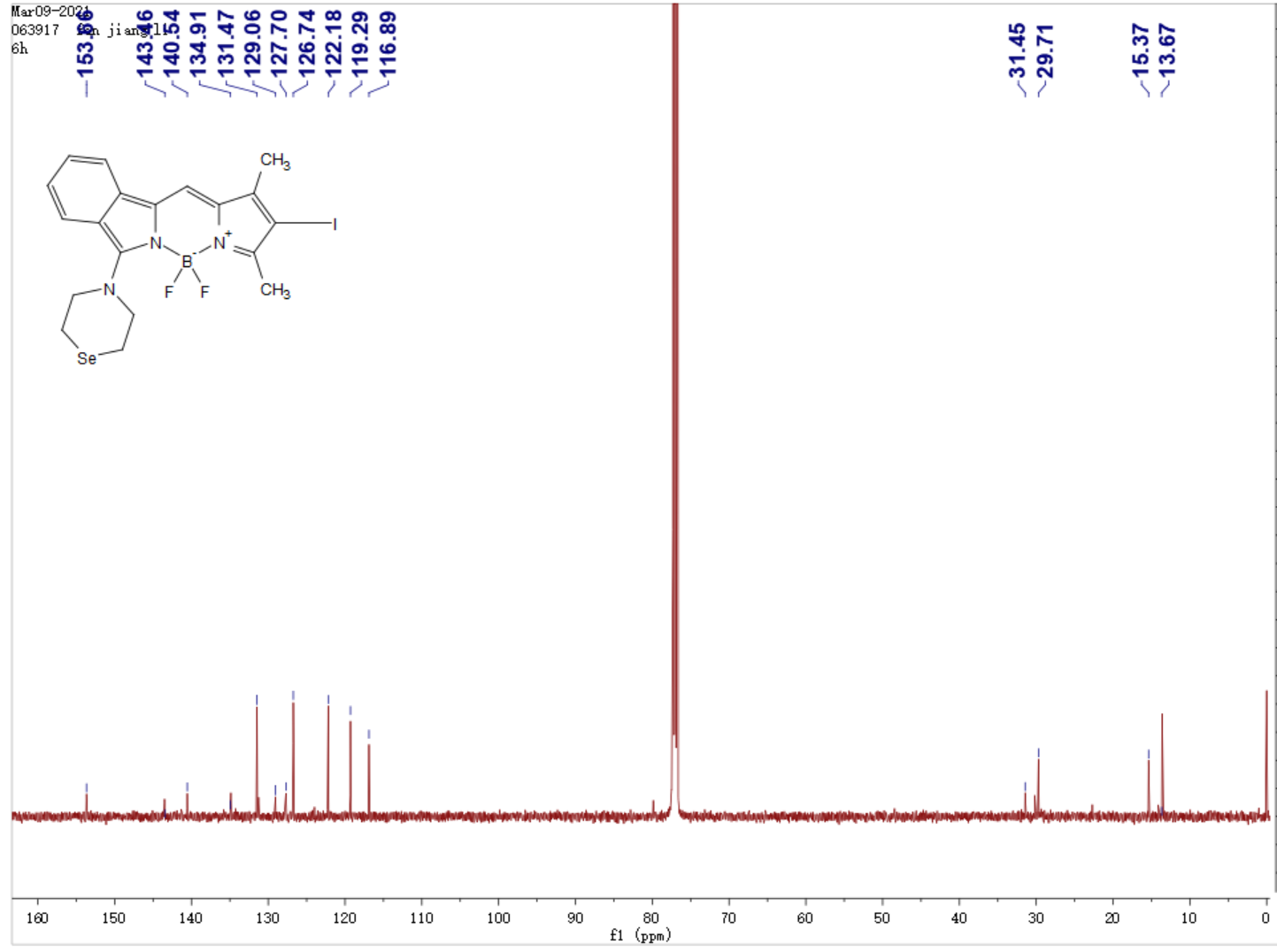


Figure S13, ^13^C-NMR spectrum of BODSeI in CDCl_3_


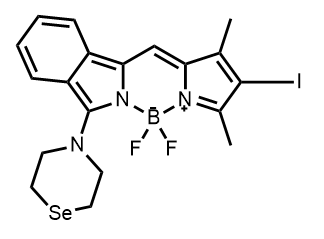


Figure S14, mass spectrum of BODSeI calcd. [M], C_19_H_20_BF_2_ N_3_Se: 544.9850, [M+Na],567.9742; found [M]:544.9851, [M+Na]:567.9746.
